# Chloroplast Genome Sequences and Comparative Analyses of Combretaceae Mangroves with Related Species

**DOI:** 10.1155/2020/5867673

**Published:** 2020-09-29

**Authors:** Ying Zhang, Hai-Li Li, Jun-Di Zhong, Yun Wang, Chang-Chun Yuan

**Affiliations:** Life Science and Technology School, Lingnan Normal University, Zhanjiang 524048, China

## Abstract

In the Combretaceae family, only two species of *Lumnitzera* and one species of *Laguncularia* belong to mangroves. Among them, *Lumnitzera littorea* (Jack) Voigt. is an endangered mangrove plant in China for the limited occurrence and seed abortion. In contrast, *Lumnitzera racemosa* Willd. is known as the most widespread mangrove plant in China. *Laguncularia racemosa* C. F. Gaertn., an exotic mangrove in China, has the fast growth and high adaptation ability. To better understand the phylogenetic positions of these mangroves in Combretaceae and in Myrtales and to provide information for studies on evolutionary adaptation for intertidal habitat, the complete chloroplast (cp) genomes of *Lu. racemosa* and *La. racemosa* were sequenced. Furthermore, we present here the results from the assembly and annotation of the two cp genomes, which were further subjected to the comparative analysis with *Lu. littorea* cp genomes we published before and other eleven closely related species within Myrtales. The chloroplast genomes of the three Combretaceae mangrove species: *Lu. littorea*, *Lu. racemosa*, and *La. racemosa* are 159,687 bp, 159,473 bp, and 158,311 bp in size. All three cp genomes host 130 genes including 85 protein-coding genes, 37 tRNAs, and 4 rRNAs. A comparative analysis of those three genomes revealed the high similarity of genes in coding-regions and conserved gene order in the IR and LSC/SSC regions. The differences between *Lumnitzera* and *Laguncularia* cp genomes are the locations of *rps19* and *rpl2* genes in the IR/SC boundary regions. Investigating the effects of selection events on shared protein-coding genes showed a relaxed selection had acted on the *ycf2*, *ycf1*, and *matK* genes of Combretaceae mangroves compared to the nonmangrove species *Eucalyptus aromaphloia*. The phylogenetic analysis based on the whole chloroplast genome sequence with one outgroup species strongly supported three Combretaceae mangroves together with other two Combretaceae species formed a cluster in Combretaceae. This study is the first report on the comparative analysis of three Combretaceae mangrove chloroplast genomes, which will provide the significant information for understanding photosynthesis and evolution in Combretaceae mangrove plants.

## 1. Introduction

Mangroves are critical marine resources for their remarkable ability to tolerate seawater and are uniquely adapted to tropical and subtropical coasts [1]. Mangrove forests played an important role in ecosystem services and supported coastal livelihoods, yet they are relatively low in the number of species [2]. In the world, the increasing number of endangered mangrove species has made a huge destruction on the global economy and environment [3, 4]. In the family Combretaceae, only two genera are typical mangrove constituents: *Lumnitzera* and *Laguncularia. Lumnitzera*, including 2 species *Lumnitzera littorea* and *Lumnitzera racemosa*, is an Asian genus most characteristic of back mangal and ranges from India and Sri Lanka, through the Malesian region to China [5]. Due to natural and human impacts, populations of the two species in this genus have been isolated, fragmented, and highly disturbed [6]. *Laguncularia* is a monotypic genus and locally dominant in the West African and Caribbean regions of tropical America [5]. In China, two *Lumnitzera* species are both native mangrove species. *Lu*. *racemosa* can be found in almost all mangrove locations with the extensive adaptation [7], but *Lu*. *littorea* occurs only on Hainan Island and has become an endangered mangrove species due to its small population size caused by seed abortion [8, 9]. The only specie *La. racemosa* from the other genus was firstly transplanted in Dongzhai harbor, Hainan, China [10]. Having the fast growing and high adaptation ability, it was used as the pioneer specie for the mangrove restoration in estuarine and coastal regions [11]. However, its invasiveness and the possibility of replacing native mangrove species have been subjects of debate in China and there are disagreements on whether *La. racemosa* should be planted [12]. Despite the obviously different adaptive capacities to the environment of those three mangrove species, *Lumnitzera* and *Laguncularia* are considered to be closely related and could have evolved from a common ancestor [5, 13].

In plants, chloroplast plays an important role in many cell functions, including photosynthesis, carbon fixation, and stress response [14], and is one of three major genetic systems [15]. A large amount of genetic information can get from the chloroplast genome to explore the occurrence, development, evolution of species, and to develop the fields of plant genomics and bioinformatics due to its self-replication mechanism and relatively independent evolution [16]. In angiosperms, the chloroplast genome was found to be a conserved quadripartite structure composing of two copies of inverted repeat (IR), one large single copy (LSC), and one small single copy (SSC) [14]. The chloroplast genome includes 120-130 genes, primarily participating in photosynthesis, transcription, and translation [17]. Recent studies have identified considerable diversity within noncoding intergenetic spacer regions, which often include important regulatory sequences [18]. Similar to the genes, the introns in chloroplast genomes of land plants are generally conserved, but the loss of introns within protein-coding genes and tRNA genes has been reported in several plant species [19]. Until now, over 3,665 plants have been sequenced, which has greatly improved chloroplast (cp) genome research (https://www.ncbi.nlm.nih.gov/genome/browse#!/plasmids/). However, there is little genetic and genomic research on plants from the family Combretaceae. As a large plant group, Combretaceae comprises over 600 species, but only few reports on sequencing cp genomes from this family. The whole cp information will help us to understand and progress the species identification, evolution, and genetic engineering in Combretaceae plants. Furthermore, only few of about 70 mangrove species have cp genome information, although the chloroplasts may play an important role for mangroves to inhabit the sea habitat [20]. Under salt stress, the obvious changes were found in the chloroplast ultrastructure, the expression of chloroplast genes, and even chloroplast proteins [21, 22].

In this study, two whole cp genomes of Combretaceae mangroves, *Lu*. *racemosa* and *La*. *racemosa*, were sequenced by using next-generation sequencing and applying a combination of de novo and reference guided assembly (*Lu*. *littorea* (MH551146) as a reference). Using a comparative genomics approach, we analyzed the characteristics of the cp genomes of the three mangrove species from Combretaceae. Nonsynonymous (Ka) and synonymous (Ks) substitution rates of conservative protein-coding genes among the three Combretaceae mangrove cp genomes were calculated to evaluate selection pressures. The coding sequences (CDSs) under selective events were also detected. Furthermore, the phylogenetic positions of three Combretaceae mangroves in Myrtales formed a clade sister to Lythraceae and Onagraceae, which will help to understand the role of natural selection in the adaptation of Combretaceae mangrove species.

## 2. Materials and Methods

### 2.1. Plant Materials, DNA Extraction, and Sequencing

Two Combretaceae mangrove samples were collected from Tielu Bay, Sanya, China (18°17′N, 109°44′E). Cp DNA was isolated from fresh leaf tissue of one individual plant of Lu. racemosa and La. racemosa separately. DNA extraction was used by purelink genomic plant DNA purification kit (Thermo Fisher, China). The extracted DNA was sent to a sequencing company TGS (Shenzhen, China) and sequenced with 350 bp pair-end reads by using an Illumina Hiseq-2000 platform according to the standard protocol at TGS.

### 2.2. De Novo Assembly, Gap Filling, and Genome Annotation

Raw reads were first filtered to obtain the high-quality clean data by removing adaptor sequences and low-quality reads with *Q* value ≤ 20. SOAP de novo 2.04 (http://soap.denovo.html) was used to perform the initial assembly and obtain the contig sequences. The software GapCloser 1.12 (http://soap.genomics.org.cn/soapdenovo.html) was used to fill the gaps in the frame sequences diagram. The whole framework maps of the cp genomes were obtained by using the reference genes of Lu. littorea (MH551146) with the methods of [16]. The cp genome sequences were annotated with CpGAVAS software with parameters that use default values [23]. The genes in the cp genome were annotated using the DOGMA program with a parameter of 40 for the identity of the coding protein and other parameters are the default values [24], and then manually corrected. Star/stop codons and intron/exon borders were edited manually after comparation with the reference. In addition, we used tRNAscan-SE v2.0 to verify the identified tRNA genes [25]. The circular cp genome map was drawn using OGDRAW [26]. Primers were designed (Table S1) to test for correct sequence assembly. PCR amplification was performed according to a previously reported procedure [27].

### 2.3. Sequence Divergence Analysis

The complete cp genomes of Lu. racemosa and La. racemosa were compared with Lu. littorea by using the mVISTA program in Shuffle-LAGAN mode [28]. We set Lu. littorea as the reference sequence for these comparisons. The borders between single-copy regions (LSC and SSC) and inverted repeats (IR) regions among three Combretaceae cp sequences were compared by using Geneious v11.0.4 software. The sequence divergences in protein-coding genes between the three Combretaceae species were evaluated by using MEGA 7 [29]. To estimate the nucleotide diversity (Pi) values of LSC, SSC, and IR regions of three cp genomes, a sliding window analysis was conducted by using DnaSP 6 [30]. The step size was set to 200 bp, the window length was 600 bp, and the Tamura 3-parameter (T92) model was selected to test the pairwise sequence divergences [31].

### 2.4. Selection Pressure Analysis

Selective pressures were analyzed for consensus protein-coding genes among four Myrtales species (Lu. littorea, Lu. racemosa, La. racemosa, and Eucalyptus aromaphloia (NC_022396.1)). Seventy-six coding sequences (CDSs) longer than 300 bp were kept for the identification of codon usage patterns and then used for the estimation of codon usage using Codon W (http://codonw.sourceforge.net) [32]. Ka/Ks value for each gene was calculated by using the Ka/Ks calculator, and the settings were listed as previous description [32].

### 2.5. Phylogenomic Analysis

Phylogenomic analysis was performed for seventeen cp genomes including three Combretaceae mangroves in this study. Multiple sequence alignment was performed by using MAFFT software [33] to obtain the aligned chloroplast genomes before constructing the phylogenetic tree. The maximum likelihood tree, neighbor-joining tree, UPGMA tree, and test maximum parsimony tree were constructed using MEGA X [34] with Rhizophora stylosa as outgroup, and a bootstrap test was set to 1000 replicates to calculate each bootstrap value.

## 3. Results and Discussion

### 3.1. Assembly and Features of cp Genomes for Three Species

**Figure 1:**
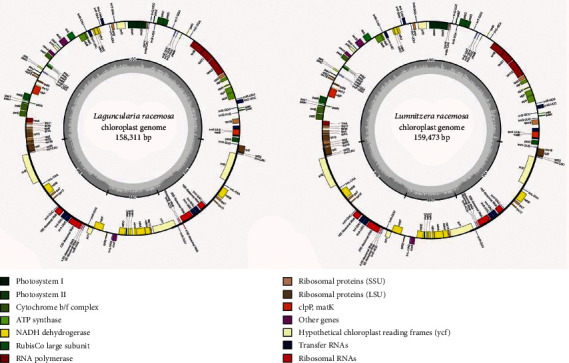
Gene maps of *Lu*. *racemosa* and *La*. *racemosa* chloroplast genomes. Genes drawn inside the circle are transcribed clockwise, and those outside are transcribed counterclockwise. Genes belonging to different functional groups are color-coded. The darker gray color in the inner circle corresponds to the GC content, and the lighter gray color corresponds to the AT content.

The complete cp genomes of *Lu*. *racemosa* and *La*. *racemosa* are 159,473 bp and 158,311 bp in length (GenBank accession number: MH551146, MH551145), respectively (Figure 1). The minor differences in length of cp genomes are no more than 214 bp in genus *Lumnitzera*. The maximum difference in length of cp genomes between the three Combretaceae mangroves is 1,376 bp (Table 1). The typical quadripartite structure of most angiosperms was found in both of the two cp genomes, which comprise a pair of IRs (26,402 bp for *Lu*. *racemosa* and 26,156 bp for *La*. *racemosa*) separated by the LSC (88,056 bp for *Lu*. *racemosa* and 87,023 bp for *La*. *racemosa*) and SSC (18,613 bp for *Lu*. *racemosa* and 18,887 bp for *La*. *racemosa*) regions (Table 1). The cp genomes of the three Combretaceae mangroves are considerable conservation in length, and a previous study showed that the IR sequence length of some species in the Myrtales is 25.7-28.7 kb [35]. The same GC content in cp genomes (36.97%) was revealed for both *Lu*. *racemosa* and *La*. *racemosa*, similar to the value reported previously for *Lu*. *littorea* cp genome [36]. A total of 130 genes including 85 protein-coding genes, 37 tRNA genes, and 8 rRNA genes were found in three cp genomes. The only difference lies in the gene number in the IR regions between the three cp genomes (Table 1). The overall genomic structure including gene number and gene order were well-conserved.

Same as observed for other angiosperms, the protein-coding genes in the cp genomes of Combretaceae mangroves include five genes encoding photosystem I components (*psaA*, *B*, *C*, *I*, and *J*) and fifteen genes related to photosystem II (*psbA*, *B*, *C*, *D*, *E*, *F*, *H*, *I*, *J*, *K*, *L*, *M*, *N*, *T*, and *Z*), nine genes encoding large ribosomal proteins (*rpl2*, *14*, *16*, *20*, *22*, *23*, *32*, *33*, and *36*), eleven genes encoding small ribosomal proteins (*rps2*, *3*, *4*, *7*, *8*, *12*, *14*, *15*, *16*, *18*, and *19*), six genes encoding ATP synthase and electron transport chain components, and eleven genes encoding NADH dehydrogenase (Table 2). For the genes having two gene copies in IRs regions, *rps19* is found in the cp genome of *La. Racemosa* and same with *Lu*. *littorea* [36], but in *Lu*. *racemosa* has only one *rps19* gene. For the genes containing an intron, *trnK-UUU*, *trnV-UAC*, and *trnG-UCC* are found in cp genome of *La*. *Racemosa*, but in *Lu*. *racemosa* no intron is found in both of them. Other gene *trnR-AGG* with one intron is found in the cp genome of *Lu*. *racemosa*, but no intron in the cp genome of *La*. *racemosa* (Tables S2 and S3). The majority of reported intron losses have been observed in specific plant groups or species including monocots, eudicots, and gymnosperms [17].

### 3.2. Comparison of cp Genomes of Three Combretaceae Mangroves

Gene content and structure are found to be conserved in the chloroplast genome sequences of most autotrophic land plants, but there are still some protein-encoding genes being absent in some specific species. *InfA*, *rpl22*, and *ndh* were the most reported genes are found from the chloroplast genomes to nuclear or mitochondrial genomes [17, 37]. But in this study, only gene *infA* was absent in the three cp genomes, and the same loss events are found in other Myrtales plants [38]. For the translation initiation factor gene, *infA* in cp has been lost independently at least 24 times in angiosperms and evidence provided from some cases suggested functional replacement by a nucleus copy [39]. The essential gene *rpl22*, which is reported in 57 chloroplast genomes to have been deleted from the chloroplast and transferred to the nuclear genome [17], is kept in three cp genomes of Combretaceae mangroves. The *ndh* proteins assemble into the photosystem I complex to mediate cyclic electron transport in chloroplasts and play an important role in facilitating chlororespiration, and in some autotrophic plants which were found to be lost in cp genomes [24]. In the three Combretaceae mangroves, all the eleven *ndh* genes in cp genomes encoding *ndh* subunits and involved in photosynthesis [40] were present, consistent with other Myrtle plants such as those in Lythraceae [40].

**Figure 2:**
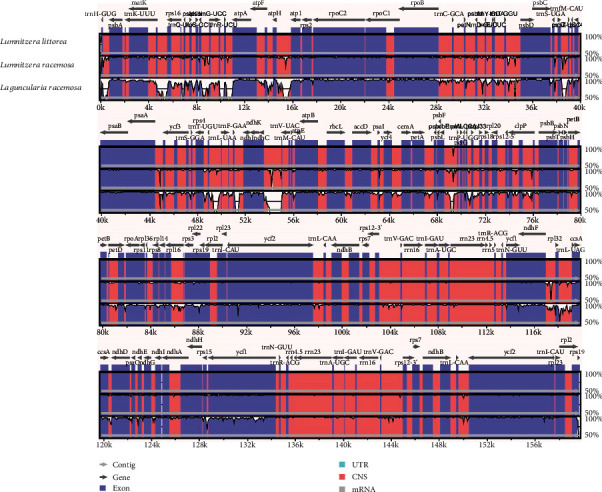
Identity plot comparing the chloroplast genomes of *Lu. littorea*, *Lu. racemosa*, and *La. racemosa*. The vertical scale indicates the percentage of identity, ranging from 50 to 100%. The horizontal axis indicates the coordinates within the chloroplast genome. Genome regions are color-coded as protein-coding, rRNA, tRNA, intron, and conserved noncoding sequences (CNS).

The whole-genome alignment revealed the high sequence similarity across the three cp genomes, suggesting that Combretaceae mangrove cp genomes are all conserved (Figure 2). All three cp genomes showed that the single-copy regions are more divergent than the IR regions as observed in other angiosperms [31], which is possibly due to error correction occurring via gene conversion between IRs [41]. Furthermore, the coding regions are more conserved than noncoding regions, as seen in other plants [31]. As found in most other green terrestrial plants [42], the maturase K (*matK*) in the cp genomes of the three Combretaceae mangrove species is also located within the *trnK* intron. *rpl2* gene is found to be a transspliced gene in the three Combretaceae cp genomes, which was observed to be intron loss in three Lagerstroemia cp genomes and considered to be one of the important evolutionary events in the Lythraceae of the Rosids [40]. The four rRNA genes and two tRNA genes of *trnI* and *trnA* are clustered as 16S-*trnA*-23S-4.5S-5S in the IR region in these three cp genomes and in most other green terrestrial plants [32, 43]. The most divergent coding regions in the three cp genomes were *ycf1*, *rps19*, and *ndhF*. In noncoding regions, the highest sequence divergence among these three cp genomes is regions *trnG-UCC*/*atpA*, *atpH*/*aptl*, *ndhC*/*trnM-CAU*, and *trnL-UAG*/*ccsA*. These hotspot regions can furnish valuable information for exploring molecular markers for phylogenetic studies and identification of Combretaceae species.

**Figure 3:**
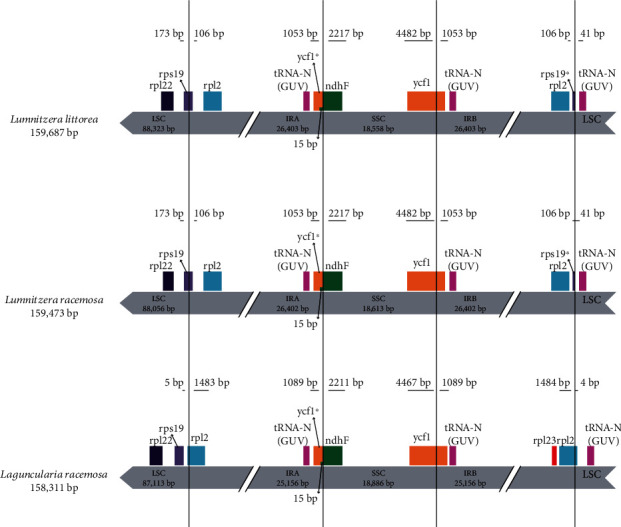
Comparison of IR, LSC, and SSC junction positions among the three chloroplast genomes. The features drawn are not to scale. The symbol ^∗^ means pseudogene created by IRb/SSC border extension into ycf1 genes and IRB/LSC border extension into *rps19* genes.

The angiosperms chloroplast genomes are highly conserved, but slightly vary as a result of either expansion or contraction of the single-copy (SC) and IR boundary regions [44]. The IR/SC junction position change caused by the expansion and contraction of IR/SC boundary regions was usually considered as a primary mechanism in creating the length variation of the higher plant cp genomes [40]. In this study, we found the IR/SC junction position change among the cp genomes between *Lumnitzera* and *Laguncularia* mangroves and the high conservation in *Lumnitzera* genus (Figure 3). The functional *ycf1* gene crossed the IRA/SSC boundary creating *ycf1* pseudogene fragment at the IRb region in all the genomes as in other land plants [44]. All the *ycf1* pseudogene overlapped with the *ndhF* gene in the SSC and IRA junctions with a stretch of 15 bp, and the *ndhF* gene is located in SSC regions in all cp genomes. Genes *rps19* crossed the IRB/LSC boundary creating *rps19* pseudogene fragment were only be found in *Lu*. *littorea* and *Lu*. *racemosa* cp genomes. However, in *La*. *racemosa*, the *rps19* gene was located in the LSC region, 5 bp apart from the IRb/LSC border. The *rpl2* genes crossed the LSC/IRB and LSC/IRA junction in *La*. *racemosa* cp genome but the distances between *rpl2* and the border is 106 bp in both of the two *Lumnitzera* species. Those variations at the IR/SC borders in these three cp genomes contribute to the differences in length of the cp genome sequence as a whole and were found in other plants [31].

### 3.3. Genome Sequence Divergence among Combretaceae Species

**Figure 4:**
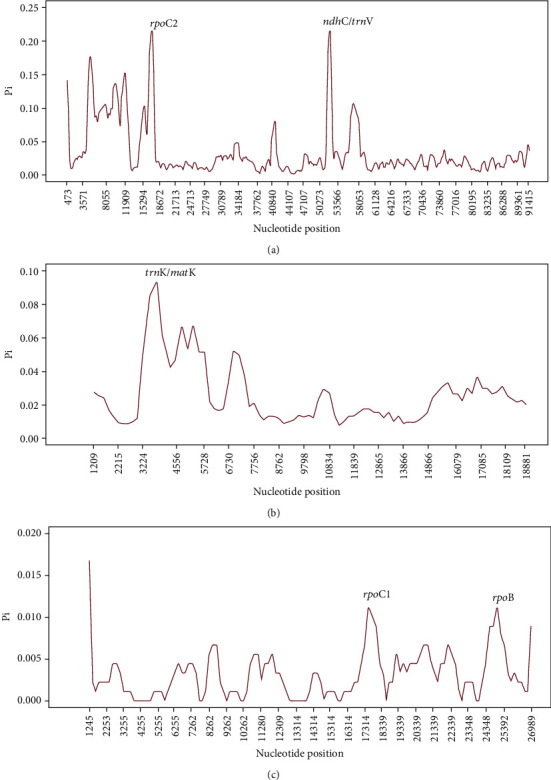
Comparative analysis of nucleotide variability (Pi) values among three Combretaceae mangrove cp genome sequences. (a) Analysis of the LSC regions; (b) Analysis of the SSC regions; (c) Analysis of the IR regions. (Window length: 600 bp, step size: 200 bp). *x*-axis: position of the midpoint of a window, *y*-axis: nucleotide diversity of each window.

The sequence divergence among the three Combretaceae mangrove cp genomes was investigated by calculating the nucleotide variability (Pi) values within 600 bp windows (200 bp stepwise moving) in LSC, SSC, and IR regions (Figure 4). In the LSC, SSC, and IR regions, the values were found to vary from 0 to 0.22 with a mean of 0.085, from 0 to 0.095 with a mean of 0.063 and from 0 to 0.0169 with a mean of 0.082 separately. These results mean conservation between the three Combretaceae mangrove cp genomes. However, the certain highly variable regions have also been found in *rpoC2* and *ndhC*/*trnV* with Pi > 0.02 in LSC regions, *trnK*/*matK* with Pi > 0.08 in SSC regions, and *rpoC1* and *rpoB* with Pi > 0.01 in IRs regions (Figure 4). All those regions or some of them have also been identified as highly variable in other plants, which showed great potential as sources of useful phylogenetic markers for Combretaceae and other plant species [31, 45].

### 3.4. Selection Events in Protein-Coding Genes

**Figure 5:**
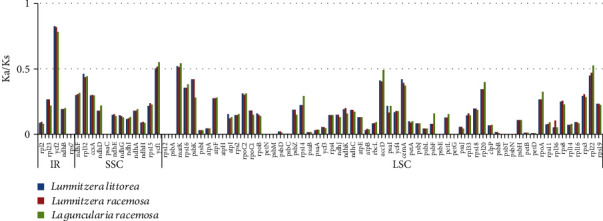
Ka/Ks rations for protein-coding genes from *Lu. littorea*, *Lu. racemosa*, and *La. racemosa* chloroplast genome in comparison with *Eucalyptus aromaphloia*.

The nonsynonymous (Ka) and synonymous (Ks) substitution rations (Ka/Ks) were calculated for 78 consensus protein-coding genes to estimate selective pressures in cp genomes of the three Combretaceae mangroves and one reference species *E. aromaphloia*, which is a normal land and nonhalophytes plant, to evaluate the selective pressure (Table S4). The average Ka/Ks ratio of the shared genes analyzed across the three cp genomes was 0.1638. Although all of the Ka/Ks values were all less than 1.0 in Figure 5, the Ka/Ks ration of three genes (*ycf1*, *ycf2*, and *matK*) in all Combretaceae mangroves were within the range of 0.5 to 1.0 indicating a relaxed selection [31]. Furthermore, there was one gene *rpl22* only found in *La. racemosa* with the Ka/Ks ration within the range of 0.5 to 1.0. Another gene *accD* with the Ka/Ks ration is closed to 0.5 in *La*. *racemosa* and not in the other two mangroves in Figure 5. The most conserved genes with Ka/Ks values of 0 in the three cp genomes were *atpH*, *petG*, *petN*, *psaC*, *psbE*, *psbM*, *psbN*, *psbT*, and *rps7*, suggesting very strong purifying selection (Table S4). For the none positive selection gene was found in Combretaceae mangroves cp genome, this may be because adaptive modifications to salt stresses targeting genes in the nucleus were sufficient to maintain homeostasis for photosynthesis since there are a variety of strategies for plants to adapt to the environment, so there is no need for adaptive evolution of chloroplast-encoded genes [46]. But genes under relative selection in cp genomes may play some roles in Combretaceae mangroves evolution and environment adaptation. *Ycf* genes have proved useful for analyzing cp genome variation in higher plants and algae, even though the function is not thoroughly known [47]. Among *Ycf* genes, *ycf1* and *ycf2* are the two largest genes and are located in IR/SC junction and IR region, respectively, almost in all plants [48]. The gene *ycf1* encodes a component of the chloroplast's inner envelope membrane protein translocation [49]. It is also highly variable in terms of phylogenetic information at the level of species, has also been shown to be subject to relative selection with *rps12* and *matK* genes in three Combretaceae mangroves, and has also been identified in many plant lineages [50]. The *ycf2* gene in the cp genome is regarded as having one of the fastest evolutionary rates within the cp genome for the lost events [51]. Even though a giant reading frame of *ycf2* is believed as unknown function in land plants, which is responsible for the differences in the competitive behavior of plastid genotypes [52]. The nucleotide sequence similarity of *ycf2* in land plant is extraordinarily low compared to other plastid-encoded genes, being less than 50% across bryophytes, ferns, and seed plants [53]. The gene *matK* (maturase K gene) is a plant chloroplast gene [54] and encodes an intron maturase involved in the cutting and splicing of Group II RNA transcriptional introns [55]. By sequence analysis, there are one well-conserved domain X and the remnants of a reverse transcriptase domain in the coding sequence of *matK* [55]. The *matK* gene is always found to be located within the intron of the *trnK* gene (Figure 1) same with some land plants [56]. *MatK* gene is identified to be essential for plant cell survival, and the expression of it needs to be tightly regulated to prevent detrimental effects and establishes another link between leaf variegation and chloroplast translation [57]. For the studies of plant systematics, the *matK* gene is the high selectively used gene as the DNA barcoding fragment for plants [58].

Gene *rpls* are ribosome genes that have been proven to be essential for the chloroplast ribosome development in plants [59]. Among them, *rpl22* is always found to have a frameshift mutation generated premature termination codons within it in land plants. In some species, *rpl22* was also found to be truncated with considerable length variation [60]. For the plastid gene, *accD*, which encodes the *β*-carboxyl transferase subunit of acetyl-CoA carboxylase, is an essential and required component for plant leaf development [61]. The same site-specific selection events of it have been observed in other plants [8], and which have been shown to affect the plant fitness by altering the acetyl-CoA carboxylase production [62]. Both genes *rpl22* and *accD* were only found to be under different selection between *La. racemosa* and two *Lumnitzera* species, and for *La. racemosa*, which is an introductive mangrove species in China and having the different original habitat other two *Lumnitzera* mangroves [5].

### 3.5. Phylogenetic Analysis of Three Combretaceae Mangrove cp Genomes and Related Myrtale Species

**Figure 6:**
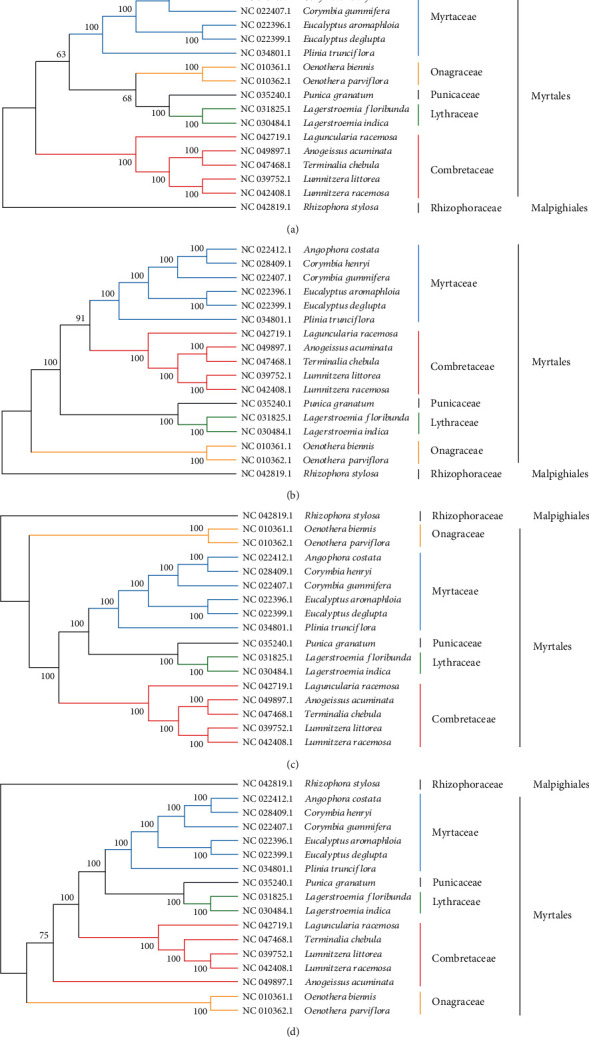
Phylogenetic relationship between five Combretaceae cp genomes and 11 related species in Myrtales, inferred from the whole cp genome sequences. Rhizophoraceae plant: Rhizophora stylosa as outgroup. (a) Maximum likelihood tree; (b) test maximum parsimony tree; (c) neighbor-joining tree; (d) UPGMA tree.

Cp genome sequences are useful for deciphering phylogenetic relationships among closely related taxa and for clarifying the evolutionary patterns of plant species [63]. To evaluate the phylogenetic position of Combretaceae mangrove species in the Myrtales, the whole chloroplast genome sequences of seven Combretaceae species with other nine plants in Myrtales were used to infer their phylogenetic relationships (Figure 6). Among those cp genomes, one mangrove plant Rhizophora stylosa was set as outgroup. By the phylogenetic analysis, 16 Myrtale species were clustered into five families, six species in Myrtaceae, two species in Onagraceae, and five Combretaceae mangroves were clustered into their own groups. Furthermore, two Lumnitzera genera were clustered into one subclade in the Combretaceae group. Within the family Combretaceae, Laguncularia and Lumnitzera are quite closely related in the field in China and are considered to be evolved from a common ancestor [5]. In this study, the position of Combretaceae mangroves are more closed with Lythraceae and Punicaceae species in Myrtales, which was showed in all the four polygenetic trees. In Myrtales, there included three significant shifts in diversification rates, one of them contributed from Combretaceae [64]. The chloroplast genome of those three Combretaceae mangroves will provide valuable and essential genetic information to further the phylogenetic resolution among angiosperms [63].

## 4. Conclusions

With the help of high-throughput sequencing technology, we comparatively analyzed the complete cp genomes of three Combretaceae mangroves. The gene contents and gene orders of the three cp genomes were highly conserved. Gene ycf1, rps19, and ndhF were found to be the most divergent coding regions, and there are still some other noncoding regions with the high sequence divergence, which could potentially serve as molecular markers in phylogenetic studies. Three genes ycf1, ycf2, and matK were found to be under relaxed selection in the cp genomes of three Combretaceae mangroves. Phylogenetic analysis showed that the position of Combretaceae mangroves is closest to Punicaceae and Lythraceae species in Myrtales.

## Figures and Tables

**Table 1:** Comparative analyses on the basic feature of the chloroplast genomes of three Combretaceae mangrove species.

	*Lu. littorea*	*Lu. racemosa*	*La. racemosa*
Length (bp)	159,687	159,473	158,311
GC content (%)	37.01	36.97	36.97
AT content (%)	62.99	63.03	63.03
LSC length (bp)	88,323	88,056	87,113
SSC length (bp)	18,558	18,613	18,886
IR length (bp)	26,403	26,402	25,156
Gene number	130	130	130
Gene number in IR regions	39	38	36
Protein-coding gene number	85	85	85
Protein-coding gene (%)	65.1	65.4	65.1
rRNA gene number	8	8	8
rRNA (%)	6.2	6.2	6.2
tRNA gene number	37	37	37
tRNA (%)	28.7	28.5	28.7

**Table 2:** Genes in the cp genomes of three Combretaceae mangrove species.

Category	Gene group	Gene name
Photosynthesis	Photosystem I	*psaA*, *psaB*, *psaC*, *psaI*, *psaJ*
	Photosystem II	*psbA*, *psbB*, *psbC*, *psbD*, *psbE*, *psbF*, *psbH*, *psbI*, *psbJ*, *psbK*, *psbL*, *psbM*, *psbN*, *psbT*, *psbZ*
	Cytochrome b/f complex	*petA*, *petB*, *petD*, *petG*, *petL*, *petN*
	ATP synthesis	*atpA*, *atpB*, *atpE*, *atpF*, *atpH*, *atpI*
	Large subunit of RuBisCo	*rbcL*
	NADH dehydrogenase	*ndhA*, *ndhB*, *ndhC*, *ndhD*, *ndhE*, *ndhF*, *ndhG*, *ndhH*, *ndhI*, *ndhJ*, *ndhK*
Self-replication	Ribosomal RNA genes	*rrn4.5*, *rrn5*, *rrn16*, *rrn23*
	Ribosomal RNA genes (SSU)	*rps2*, *rps3*, *rps4*, *rps7*, *rps8*, *rps12*^∗^, *rps14*, *rps15*, *rps16*, *rps18*, *rps19*
	Ribosomal RNA genes (LSU)	*rpl2*, *rpl14*, *rpl16*, *rpl20*, *rpl22*, *rpl23*, *rpl32*, *rpl33*, *rpl36*
	RNA polymerase	*rpoA*, *rpoB*, *rpoC1*, *rpoC2*
	Transfer RNA genes	*trnA-UGC*, *trnC-GCA*, *trnD-GUC*, *trnE-UUC*, *trnF-GAA*, *trnfM-CAU*, *trnG-UCC*, *trnH-GUG*, *trnI-CAU*, *trnI-GAU*, *trnK-UUU*, *trnL-CAA*, *trnL-UAA*, *trnL-UAG*, *trnM-CAU*, *trnN-GUU*, *trnP-UGG*, *trnQ-UUG*, *trnR-ACG*, *trnR-UCU*, *trnS-GCU*, *trnS-GGA*, *trnS-UGA*, *trnT-GGU*, *trnT-UGU*, *trnV-GAC*, *trnV-UAC*, *trnW-CCA*, *trnY-GUA*
Other genes	Maturase	*matK*
	Envelope membrane factor	*cemA*
	Subunit of acetyl-CoA	*accD*
	C-type cytochrome synthesis gene	*ccsA*
	Protease	*clpP*
	Hypothetical chloroplast reading	*ycf1*, *ycf2*, *ycf3*, *ycf4*

^∗^Pseudogene.

## Data Availability

The analysis and experimental data used to support the findings of this study are included within the article.

## Supplementary Materials

Supplementary Materials The following are available online at https://www.mdpi.com/xxx/s1. Table S1: primers used for gap closing and sequencing verification in *Laguncularia racemosa* and *Lumnitzera racemosa*. Table S2: functional classification of *Lumnitzera racemosa* chloroplast genome genes. Table S3: functional classification of *Laguncularia racemosa* chloroplast genome genes. Table S4: Ka/Ks value of the consensus protein-coding genes in three cp genomes of Combretaceae mangroves.
