# Supplementary material for: Chloroplast Genome Sequences and Comparative Analyses of Combretaceae Mangroves with Related Species
Source: Biomed Res Int. 2020 Sep 29;2020:5867673. doi: 10.1155/2020/5867673 (PMC7545412; doi:10.1155/2020/5867673)
Supplement: Supplementary Materials — The following are available online at https://www.mdpi.com/xxx/s1. Table S1: primers used for gap closing and sequencing verification in Laguncularia racemosa and Lumnitzera racemosa. Table S2: functional classification of Lumnitzera racemosa chloroplast genome genes. Table S3: functional classification of Laguncularia racemosa chloroplast genome genes. Table S4: Ka/Ks value of the consensus protein-coding genes in three cp genomes of Combretaceae mangroves. [file 5867673.f1.doc]

*Table S1. Primers used for gap closing and sequencing verification in Laguncularia racemosa and Luminitzera recemosa*

| Species | *Laguncularia racemosa* | | |
| --- | --- | --- | --- |
| Number | Primers | Sequence | Information |
| 1 | 794-F | GGTTGAAAGCCATAGTAC | Gap Closing |
|  | 1,314-R | GCTTGTTACATGGGTCGTG |  |
| 2 | 6,252-F | CCATAACATGCCTCTAGTTT | Gap Closing |
|  | 8,047-R | CTAAAGATATTAAGCATAAC |  |
| 3 | 27,253-F | GAGATCCGCTACAGAACG | Gap Closing |
|  | 27,990-R | CATATTCTTCTGAATTATA |  |
| 4 | 30,832-F | GATTCGAAGAAATTGCCAG | Gap Closing |
|  | 31,120-R | CCAATGGAAACTTAAGAG |  |
| 5 | 112,090-F | GGAAAGTTCAAAAATCAT | Gap Closing |
|  | 113,239-R | CATAATAAATATTTCTCTTC |  |
| 6 | 120,986-F | CAACTGTATTCAAAGTGT | Gap Closing |
|  | 121,428-R | GTATCTTGGCCCAATCTC |  |
| 7 | 141,985-F | GGCTCGAACTGATGACTTC | Gap Closing |
|  | 142,493-R | GTCTCAAAGGCGCGCGG |  |
| 8 | 150,667-F | CAAGATCTCGTACATTGG | Gap Closing |
|  | 150,969-R | CTGGATGTGAATGAGTCG |  |
| Species | *Luminitzera recemosa* | | |
| Number | Primers | Sequence | Information |
| 1 | 18,396-F | CAAGAATTCTTGTTATATG | Gap Closing |
|  | 18,522-R | CCGGTGATATAACGCAG |  |
| 2  3  4  5  6  7 | 42,177-F  42,800-R  94,204-F  94,373-R  118,070-F  118,207-R  123,237-F  126,393-R  144,762-F  144,932-R  159,122-F  159,421-R | GGCTCTAGGCTGAGTAGCA  GTGGTGATTTAGTGGCAG  CAGATATATCATGGCGAATTC  CGAACATTTGGAGATCTC  CATTTAGTTCGGTATTCG  GATCAATCTTCCGTTAC  GGTTTAAACGAATCGCACG  GTATTGCTCCCGTTGGAC  CTCATGTACGGTTCTGTAG  GTTCTTGTGAATTATGGCC  CGGTAGTAAGAGGAGTAG  GTTCTTAATAAATGATTCGC | Gap Closing  Gap Closing  Gap Closing  Gap Closing  Gap Closing  Gap Closing |

*Table S2. Functional classification of Lumitzera racemosa chloroplast genome genes*

| Category | Gene group | Gene name |
| --- | --- | --- |
| Genes for photosynthesis | Photosystem I | *psa*A, *psa*B, *psa*C, *psa*I, *psa*J |
|  | Photosystem II | *psbA, psbB, psbC, psbD, psbE, psbF, psbH, psbI, psbJ, psbK, psbL, psbM, psbN, psbT, psbZ* |
|  | Cytochrome b/f complex | *petA, petB^b^, petD^b^, petG, petL, petN* |
|  | ATP synthesis | *atpA, atpB, atpE, atpF^b^, atpH, atpI* |
|  | Large subunit of RuBisCo | *rbcL* |
|  | NADH dehydrogenase | *ndhA^b^, B^ab^, C, D, E, F, G, H, I, J, K* |
| Self-replication | Ribosomal RNA genes | *rrn4.5^a^, rrn5^a^, rrn16^a^, rrn23^a^* |
|  | Ribosomal RNA genes (SSU) | *rps2, rps3, rps4, rps7^a^, rps8, rps12^d^, rps14, rps15, rps16, rps18, rps19 ^a^* |
|  | Ribosomal RNA genes (LSU) | *rpl2^ab^, rpl14, rpl16^b^, rpl20, rpl22, rpl23^a^, rpl32, rpl33, rpl36* |
|  | RNA polymerase | *rpoA, B, C1^b^, C2* |
|  | Transfer RNA genes | *trnA-UGC^ab^, trnC-GCA, trnD-GUC, trnE-UUC, trnF-GAA, trnfM-CAU, trnG-UCC^a^, trnH-GUG, trnI-CAU^a^, trnI-GAU^ab^, trnK-UUU, trnL-CAA^a^, trnL-UAA, trnL-UAG, trnM-CAU, trnN-GUU^a^, trnP-UGG, trnQ-UUG, trnR-ACG^ab^, trnR-UCU, trnS-GCU, trnS-GGA, trnS-UGA, trnT-GGU, trnT-UGU, trnV-GAC^a^, trnV-UAC, trnW-CCA, trnY-GUA* |
| Other genes | Maturase | *matK* |
|  | Envolope membrane factor | *cemA* |
|  | Subunit of acetyl-CoA | *accD* |
|  | C-type cytochrome synthesis gene | *ccsA* |
|  | Protease | *clpP^b^* |
|  | Hypothetical chloroplast reading | *ycf1^a^, ycf2^a^, ycf3^b^,ycf4* |

(a)Two gene copies in Irs; (b) gene containing a single intron; (c) gene containing two introns; (d) pseudogene

Table S3. Functional classification of *Laguncularia racemosa* chloroplast genome genes

| Category | Gene group | Gene name |
| --- | --- | --- |
| Genes for photosynthesis | Photosystem I | *psaA, psaB, psaC, psaI, psaJ* |
|  | Photosystem II | *psbA, psbB, psbC, psbD, psbE, psbF, psbH, psbI, psbJ, psbK, psbL, psbM, psbN, psbT, psbZ* |
|  | Cytochrome b/f complex | *petA, petB^b^, petD^b^, petG, petL, petN* |
|  | ATP synthesis | *atpA, atpB, atpE, atpF^b^, atpH, atpI* |
|  | Large subunit of RuBisCo | *rbcL* |
|  | NADH dehydrogenase | *ndhAb, B^ab^, C, D, E, F, G, H, I, J, K* |
| Self-replication | Ribosomal RNA genes | *rrn4.5^a^, rrn5^a^, rrn16^a^, rrn23^a^* |
|  | Ribosomal RNA genes (SSU) | *rps2, rps3, rps4, rps7^a^, rps8, rps12^d^, rps14, rps15, rps16^b^, rps18, rps19* |
|  | Ribosomal RNA genes (LSU) | *rpl2^ab^, rpl14, rpl16^b^, rpl20, rpl22, rpl23^a^, rpl32, rpl33, rpl36* |
|  | RNA polymerase | *rpoA, B, C1^b^, C2* |
|  | Transfer RNA genes | *trnA-UGC^ab^, trnC-GCA, trnD-GUC, trnE-UUC, trnF-GAA, trnfM-CAU, trnG-UCC^ab^, trnH-GUG, trnI-CAU^a^, trnI-GAU^ab^, trnK-UUU^b^, trnL-CAA^a^, trnL-UAA^b^, trnL-UAG, trnM-CAU, trnN-GUU^a^, trnP-UGG, trnQ-UUG, trnR-ACG^a^, trnR-UCU, trnS-GCU, trnS-GGA, trnS-UGA, trnT-GGU, trnT-UGU, trnV-GAC^a^, trnV-UAC^b^, trnW-CCA, trnY-GUA* |
| Other genes | Maturase | *matK* |
|  | Envolope membrane factor | *cemA* |
|  | Subunit of acetyl-CoA | *accD* |
|  | C-type cytochrome synthesis gene | *ccsA* |
|  | Protease | *clpP^b^* |
|  | Hypothetical chloroplast reading | *ycf1^a^, ycf2^a^, ycf3^b^,ycf4* |

(a)Two gene copies in Irs; (b) gene containing a single intron; (c) gene containing two introns; (d) pseudogene

Table S4. Ka/Ks value of the consensus protein-coding genes in three cp genomes of Combretaceae mangroves

| Gene | Ka/Ks | | |
| --- | --- | --- | --- |
|  | Lumnitzera littorea | Lumitzera racemosa | Laguncularia racemosa |
| *accD* | 0.415439 | 0.405901 | 0.493392 |
| *atpA* | 0.0470797 | 0.0470797 | 0.0464397 |
| *atpB* | 0.0383664 | 0.0454659 | 0.0388647 |
| *atpE* | 0.132719 | 0.132719 | 0.135008 |
| *atpF* | 0.27472 | 0.27472 | 0.279145 |
| *atpH* | 0 | 0 | 0 |
| *atpI* | 0.156412 | 0.121438 | 0.133967 |
| *ccsA* | 0.299249 | 0.301736 | 0.298898 |
| *cemA* | 0.422051 | 0.395618 | 0.373666 |
| *clpP* | 0.0692115 | 0.0692115 | 0.0759421 |
| *matK* | 0.52427 | 0.514413 | 0.540264 |
| *ndhA* | 0.183965 | 0.183775 | 0.19451 |
| *ndhB* | 0.192145 | 0.192145 | 0.205347 |
| *ndhC* | 0.187687 | 0.187687 | 0.176405 |
| *ndhD* | 0.184254 | 0.184288 | 0.218988 |
| *ndhE* | 0.149916 | 0.154426 | 0.139705 |
| *ndhF* | 0.303196 | 0.307692 | 0.316793 |
| *ndhG* | 0.143647 | 0.143194 | 0.131123 |
| *ndhH* | 0.0936022 | 0.09517 | 0.0906294 |
| *ndhI* | 0.120649 | 0.123669 | 0.134003 |
| *ndhJ* | 0.151166 | 0.151166 | 0.135581 |
| *ndhK* | 0.192877 | 0.199752 | 0.161896 |
| *petA* | 0.100583 | 0.0922767 | 0.0988582 |
| *petB* | 0.0147109 | 0.0152196 | 0.0147358 |
| *petD* | 0.00897381 | 0.00897381 | 0.00976601 |
| *petG* | 0 | 0 | 0 |
| *petL* | 0.128493 | 0.128493 | 0.155755 |
| *petN* | 0 | 0 | 0 |
| *psaA* | 0.0348083 | 0.0356369 | 0.0385352 |
| *psaB* | 0.0182465 | 0.0185902 | 0.0182606 |
| *psaC* | 0 | 0 | 0 |
| *psaI* | 0.219243 | 0.16992 | 0.219243 |
| *psaJ* | 0.0607316 | 0.0607316 | 0.0465321 |
| *psbA* | 0.00587794 | 0.00587794 | 0.00692013 |
| *psbB* | 0.0181584 | 0.0184213 | 0.014727 |
| *psbC* | 0.00838361 | 0.00838361 | 0.00535182 |
| *psbD* | 0.020933 | 0.020933 | 0.0131734 |
| *psbE* | 0 | 0 | 0 |
| *psbF* | 0.0807898 | 0.0807898 | 0.161521 |
| *psbH* | 0.112754 | 0.112754 | 0.112754 |
| *psbI* | 0.0318378 | 0.0318378 | 0.0318378 |
| *psbJ* | 0.0838691 | 0.0838691 | 0.0838691 |
| *psbK* | 0.423352 | 0.423352 | 0.284265 |
| *psbL* | 0.0467174 | 0.0467174 | 0.0467174 |
| *psbM*  *psbN*  *psbT* | 0  0  0 | 0  0  0 | 0  0  0 |
| *psbZ* | 0.190458 | 0.190458 | 0.152419 |
| *rbcL* | 0.0852198 | 0.0891717 | 0.0983729 |
| *rpl14* | 0.0758613 | 0.0758613 | 0.0804835 |
| *rpl16* | 0.0958731 | 0.0919171 | 0.0835374 |
| *rpl20* | 0.348081 | 0.348081 | 0.397465 |
| *rpl2* | 0.0903722 | 0.0963433 | 0.0824223 |
| *rpl22* | 0.45136 | 0.468876 | 0.525277 |
| *rpl23* | 0.270173 | 0.270173 | 0.2225 |
| *rpl32* | 0.463903 | 0.439072 | 0.44422 |
| *rpl33* | 0.144195 | 0.159033 | 0.146534 |
| *rpl36* | 0.0541599 | 0.10812 | 0.0540342 |
| *rpoA* | 0.267273 | 0.267861 | 0.32266 |
| *rpoB* | 0.15928 | 0.152717 | 0.141181 |
| *rpoC1* | 0.181541 | 0.181541 | 0.151387 |
| *rpoC2* | 0.313613 | 0.306595 | 0.3123 |
| *rps11* | 0.0763813 | 0.081936 | 0.0919153 |
| *rps14* | 0.222088 | 0.222088 | 0.29389 |
| *rps15* | 0.22168 | 0.239019 | 0.232438 |
| *rps16* | 0.357714 | 0.357714 | 0.385903 |
| *rps18* | 0.198142 | 0.198142 | 0.185161 |
| *rps19* | 0.236555 | 0.236555 | 0.226218 |
| *rps2* | 0.148218 | 0.148726 | 0.155836 |
| *rps3* | 0.294594 | 0.303998 | 0.286769 |
| *rps4* | 0.147396 | 0.147396 | 0.147396 |
| *rps7* | 0 | 0 | 0 |
| *rps8* | 0.255121 | 0.257276 | 0.231535 |
| *ycf1* | 0.503181 | 0.517884 | 0.551193 |
| *ycf2* | 0.825018 | 0.819694 | 0.782173 |
| *ycf3* | 0.054029 | 0.054058 | 0.0505317 |
| *ycf4* | 0.172504 | 0.179317 | 0.177318 |
